# Role of Adipokines and Other Inflammatory Mediators in Gestational Diabetes Mellitus and Previous Gestational Diabetes Mellitus

**DOI:** 10.1155/2012/549748

**Published:** 2012-04-09

**Authors:** Nikolaos Vrachnis, Panagiotis Belitsos, Stavros Sifakis, Konstantinos Dafopoulos, Charalambos Siristatidis, Kalliopi I. Pappa, Zoe Iliodromiti

**Affiliations:** ^1^2nd Department of Obstetrics and Gynecology, University of Athens Medical School, Aretaieio Hospital, 11528 Athens, Greece; ^2^Department of Obstetrics and Gynaecology, General Hospital of Chalkida, Evia, Chalkida 34100, Greece; ^3^Department of Obstetrics and Gynaecology, University Hospital of Heraklion, 71110 Heraklio, Crete, Greece; ^4^Department of Obstetrics and Gynaecology, Medical School, University of Thessaly, 41334 Larissa, Greece; ^5^3rd Department of Obstetrics and Gynecology, University of Athens, 12462 Athens, Greece; ^6^1st Department of Obstetrics and Gynecology, University of Athens, School of Medicine, Athens, Greece

## Abstract

Previous Gestational Diabetes Mellitus (pGDM) is a common condition and has been associated with future development of Type 2 Diabetes Mellitus (T2DM) and Metabolic Syndrome (MS) in women affected. The pathogenesis and risk factors implicated in the development of these conditions later in the lives of women with pGDM are not as yet fully understood. Research has recently focused on a group of substances produced mainly by adipose tissue called adipokines, this group including, among others, adiponectin, leptin, Retinol-Binding Protein-4 (RBP-4), and resistin. These substances as well as other inflammatory mediators (CRP, IL-6, PAI-1, TNF-**α**) seem to play an important role in glucose tolerance and insulin sensitivity dysregulation in women with pGDM. We summarize the data available on the role of these molecules.

## 1. Introduction

Pregnancy is a progressively hyperglycemic period of life, accompanied by increasing insulin resistance as from mid-gestation, with the hyperglycemia serving a highly important role in the nutrition and development of the fetus by providing it with adequate levels of glucose [1].

Gestational Diabetes Mellitus is a common pathologic state that increases the incidence of complications in both the mother and the fetus [2]. Furthermore, GDM and gestational dysregulation of blood glucose levels expose the women affected to higher risk for subsequent development of type 2 diabetes mellitus and cardiovascular disease later in their lives [3–5], the risk being proportional to the degree of the dysregulation.

Glucose tolerance and metabolism as well as insulin resistance are altered in Type II Diabetes Mellitus (T2DM), Gestational Diabetes Mellitus (GDM), and the postpartum period of a pregnancy complicated by pGDM. T2DM and pGDM have the same predisposing factors, namely, high body mass index before pregnancy, elevated levels of fasting glucose, and a degree of hyperglycemia in pregnancy, these leading to dysglycemia 1 to 4 months after delivery and recurrent gestational diabetes mellitus [6–26].

Although the pathophysiologic mechanisms responsible for these changes are not as yet completely understood, growing insight into the processes involved has been gained over the last few years. There are two main pathways leading to GDM, T2DM, and possibly pGDM: insulin resistance and chronic subclinical inflammation.

Insulin resistance is caused by the inability of tissues to respond to insulin and the deficient secretion of insulin by pancreatic beta cells [27–29]. The deficient secretion cannot compensate for the pregnancy-induced insulin resistance, this resulting in GDM, a condition which sometimes persists after delivery [8–12, 30].

With regard to the contribution of inflammatory processes to the pathogenesis of dysglycemia conditions, it has been reported that long-term activation of the acute phase inflammatory response is a risk factor for T2DM and cardiovascular disease [31].

Furthermore, obesity has a role in the development of both T2DM and GDM through chronic subclinical inflammation, low-grade activation of the acute phase response, and dysregulation of adipokines [31–33]. Increased levels of inflammatory agents during and after pregnancy have been reported in patients with GDM, while increased body fat has been strongly associated with inflammation and adipocyte necrosis, hypoxia, and release of chemokines which cause macrophages to infiltrate adipose tissue. Macrophages secrete cytokines which activate the subsequent secretion of inflammation mediating agents, specifically interleukin-6 (IL-6) and C-reactive protein (CRP). Moreover, other molecules such as Plasminogen Activator Inhibitor 1 (PAI-1) and sialic acid lead to dysregulations of metabolism, hyperglycemia, insulin resistance, and, finally, overt T2DM [31, 34–46].

Crucially, hormones produced by the placenta [47] and increased maternal fat mass [48] have been reported to play a major role in GDM. In this context, the gaps in the mechanisms underlying glucose metabolism in pregnancy and nonpregnancy states have initiated research efforts to uncover other potential mediators of insulin resistance, namely, the adipokines. These are a group of substances, knowledge about which is continuously expanding, that are produced mainly in the adipose tissue [49]. The group includes leptin, adiponectin, tumor necrosis factor alpha (TNF-*α*), retinol-binding protein-4 (RBP-4), resistin, visfatin, and apelin. These molecules are involved in a wide range of physiological processes including lipid metabolism, atherosclerosis, blood pressure regulation, insulin sensitivity, and angiogenesis, while they also influence immunity and inflammation. Their levels in pathologic states appear increased, with the exception of adiponectin which shows decreased levels.

In this paper we summarize the data available concerning the role of these mediators in women with pGDM.

## 2. Adipokines in pGDM

Adipokines, whose significant role in the pathogenesis of numerous pathologic conditions has recently been recognized, are adipose tissue-derived substances mediating communication and endocrine function between this metabolically active tissue and other sites throughout the body. A summary of the levels of adipokines and other inflammatory mediators in cases of pGDM is shown in Table 1.

### 2.1. Adiponectin

Adiponectin, a well-studied protein, is secreted by adipose tissue. It has insulin-sensitizing action, stimulating glucose uptake in skeletal muscle, and reduces hepatic glucose production through AMP-activated protein kinase [50], while it also possesses antiatherogenic and anti-inflammatory properties [51, 52]. The levels of adiponectin decrease as visceral fat increases [53–56] in such conditions as central obesity, insulin resistance, and diabetes mellitus; lower adiponectin levels have notably been associated with subclinical inflammation [43]. It has been shown that adiponectin levels begin to decrease early in the pathogenesis of diabetes, as adipose tissue increases in tandem with reduction in insulin sensitivity [57]. Hypoadiponectinemia has also been associated with beta cell dysfunction [58, 59], while it has additionally been linked to future development of insulin resistance [60] and type 2 diabetes mellitus [61–64], in the development of which adiponectin appears to have a causative role.

Adiponectin has also been studied in animal experiments in which it was demonstrated that it can reduce insulin resistance and enhance the action of insulin in liver, resulting in lowering of glucose blood levels [65–67]. In normal human pregnancy, adiponectin levels have been found to be unchanged or decreased [68–72] and negatively correlated with maternal BMI and insulin sensitivity [73].

In GDM pregnancies adiponectin appears to be decreased independently of maternal body mass index (BMI) or insulin sensitivity [68, 74–85]. Additionally, the fact that low circulating levels of adiponectin are found early in pregnancy has been associated with subsequent development of GDM [81]. Meanwhile, low levels of adiponectin in pregnancy, which have incidentally been associated with certain ethnic groups such as women of South Asian origin, have a significant impact on the development of GDM [86–88].

While hypoadiponectinemia is strongly associated with beta-cell dysfunction in pregnancy [89], the levels of adiponectin after delivery have been investigated in only a few studies. In one of them the investigators measured adiponectin levels in 89 women with pGDM at both 3 and 12 months postpartum and compared the values obtained with normal controls (women with normal pregnancies). They found that adiponectin levels were lower in women with pGDM at 3 months postpartum [43]. This registered decrease remains valid even after correction for body fat mass (BFM). The lower values of adiponectin are also associated with decreased insulin sensitivity and low HDL. It is of note that plasma adiponectin further decreased by 10% after 1 year in insulin-resistant women with pGDM.

Other investigators [90] studying 25 women with pGDM and comparing their adiponectin levels with those of 23 women with normal pregnancies at least 1 year after the index pregnancy (mean 4 years) found that the levels were significantly lower in women with pGDM compared to normal controls. The difference remained significant even after adjustment for BMI. This study also showed that adiponectin was negatively correlated to other inflammatory markers, namely, CRP, PAI-1, and IL-6, the which correlations remained unchanged even after adjustment for BMI.

In another study, 140 women with previous GDM and various states of glucose tolerance after delivery (8 with diabetes mellitus, 60 with impaired glucose tolerance and 72 with normal glucose tolerance) were studied and compared with 17 women with normal pregnancies [91]. The authors reported lower adiponectin levels in the women with pGDM 1.57 years postdelivery, while it is also of interest that the levels of adiponectin were progressively lower, the postpartum glucose tolerance values ranging from normality to impaired glucose tolerance and finally Diabetes Mellitus after GDM (*P* for linearity = 0.006). Plasma adiponectin was moreover negatively correlated with fasting glucose, fasting insulin, and RBP-4 levels.

Another recent study examined 60 women with GDM at 30 weeks of gestation and 6 weeks and 6 months postpartum and compared the results with normal pregnancies [92]. They did not find significantly different levels of adiponectin during and after pregnancy in the group of GDM women, but they did observe a significant difference in the group of non-GDM women (both at 6 weeks and 6 months postpartum, *P* < 0.01).

### 2.2. Leptin

Leptin is an adipokine that is produced by the ob (obese) gene in adipose tissue cells, especially in white adipose tissue, its action at the hypothalamus resulting in decreasing food intake and increasing energy consumption [93, 94]. It also regulates endocrine function, inflammation, immune response, and angiogenesis.

Its mechanism of action is to increase insulin sensitivity by influencing insulin secretion, glucose utilization, glycogen synthesis, and fatty acid metabolism [93–95]. Furthermore, it adjusts gonadotropin releasing-hormone secretion from the hypothalamus and activates the sympathetic nervous system.

In normal pregnancy, leptin concentration increases from early pregnancy onwards and decreases to normal pre-pregnancy levels before delivery [96–99]. This initial increase may be due to placental synthesis, since it occurs before the rise in maternal BMI and rapidly falls after delivery [99]. The function of increased maternal leptin is to enhance the mobilization of maternal fat stores thus enabling access of lipid substrates to the fetus [100].

In GDM, leptin has a more debatable role, since it appears to be elevated in women with GDM [101–103] but, after adjustment for BMI and insulin resistance [104, 105], it is shown to be decreased or even unaltered [102], while it has also been associated with insulin resistance in pregnancy [101, 103, 105].

There is to date an insufficient number of studies examining the role of leptin in the postpartum period following a GDM pregnancy. In one study, 89 women with pGDM were followed and found to have significantly increased plasma leptin at 3 months after delivery (*P* < 0.003) compared to controls [43]. Leptin levels were negatively associated with adiponectin but this association was not significant after the adjustment for BFM. Meanwhile, other authors have failed to find any difference in leptin levels between pGDM and normal pregnancies 18 months after delivery [91].

Leptin levels were studied [92] during pregnancy and 6 weeks and 6 months after delivery both in normal pregnancies and in pregnancies complicated by GDM. There were significantly higher levels of leptin in previous GDM pregnancies compared with normal pregnancies both at 6 weeks and 6 months postpartum.

### 2.3. Retinol-Binding Protein-4

RBP-4, which is an adipokine synthesized in hepatocytes and adipocytes, serves as a carrier for retinol in blood and is postulated to play a role in regulating glucose metabolism and insulin sensitivity [122, 123].

In pathologic glucose tolerance states (such as obesity, insulin resistance, polycystic ovary syndrome, and cardiovascular disease), RBP-4 concentration has been shown to be elevated [106, 124–128]. Other studies have reported low levels of the protein in humans with T2DM and have determined that RBP-4 concentration does not relate to insulin sensitivity in calorie restricted obese individuals [129–131]. It has moreover been demonstrated that overexpression of RBP-4 in normal mice increases insulin resistance, whereas genetic disruption of this adipokine increases insulin sensitivity [123].

In normal pregnancy, RBP-4 increases significantly between early and late pregnancy with a parallel decrease in insulin sensitivity [132], although other authors have reported a decrease in the levels of this hormone after early gestation [133].

GDM has been associated with increased, decreased, or even stable levels of RBP-4 [134–138]. In pGDM it has been shown that RBP-4 was significantly higher more than 18 months postpartum in women with normal or impaired glucose tolerance or with diabetes mellitus in the postpartum period compared with women without GDM. A trend was also documented of increasing RBP-4 values from normality to DM in the pGDM group (*P* for linearity = 0.006). Furthermore, RBP-4 was positively correlated with fasting insulin, whereas the correlation to adiponectin was negative. Finally, RBP-4 concentration was significantly higher in women with metabolic syndrome than in those without [91].

Another study [92] that measured RPB-4 levels in 60 women with GDM during pregnancy as well as 6 weeks and 6 months after delivery found a positive correlation of RBP-4 with fasting insulin levels. Additionally, there was a significant reduction in RBP-4 in the control group between delivery and 6 weeks and 6 months postpartum, although there was no respective decrease in the GDM group. Between the two groups, a significant difference in the levels of RBP-4 (*P* < 0.05) was not observed until 6 weeks postpartum.

### 2.4. Resistin

Resistin, a hormone expressed by adipocytes as well as monocytes and macrophages [107], appears to have levels that parallel the mass of adipose tissue [107–109]. In obesity and insulin resistance, the role of resistin is as yet highly controversial [110, 111], although its function has been associated with impaired glucose tolerance [106, 111]. The results in animal experiments are indicative of insulin resistance induction in animals, but the same is not true in humans [107, 111–113].

In normal pregnancy it is expressed in human placenta [114], with plasma resistin levels in pregnant women being significantly higher as compared to normal controls. It increases in the third trimester [68, 76, 114–116] and may regulate energy metabolism during pregnancy.

In GDM its levels have been found either elevated or decreased [76, 117–120], with some studies having reported elevated maternal resistin in GDM [68, 118, 121, 147], while others found lower [73, 118] or unaltered values [70].

With regard to pGDM, one study [91] showed that resistin was significantly higher 18 months postpartum in women with normal or impaired glucose tolerance who had pGDM compared to a group of women who did not develop DM during pregnancy. This could be explained by a biphasic effect of insulin on the release of resistin, whereby low concentration of insulin increases the release of resistin, while this is reduced at higher insulin levels [76]. The authors also reported that plasma resistin levels correlated with BMI, fat quantity, and plasma insulin and that the presence of metabolic syndrome was not significantly associated with plasma resistin levels in the postpartum period.

### 2.5. Visfatin

Visfatin is an adipocytokine produced mainly in visceral fat, as compared to subcutaneous fat, which exerts insulin mimetic action [139] and, additionally, plays a pro-inflammatory role [140].

It has higher concentrations in cases of obesity or insulin resistance, including T2DM and metabolic syndrome [139, 141, 142]. Conversely, other studies showed no relation of visfatin to insulin sensitivity or increased BMI and visceral fat mass [143, 144].

In pregnancy, although this hormone has been reported by some authors to maintain the same levels in the third trimester as in the non-pregnant state [145], other studies have demonstrated an increase [72, 146]. Visfatin levels peak between 19 and 26 weeks of gestation, while at between 27 and 34 weeks visfatin has the lowest serum concentrations [147].

Morgan et al. reported that visfatin may have a paracrine or autocrine action since it is locally increased in omental fat without increased plasma levels in pregnancy [148].

**Figure 1:**
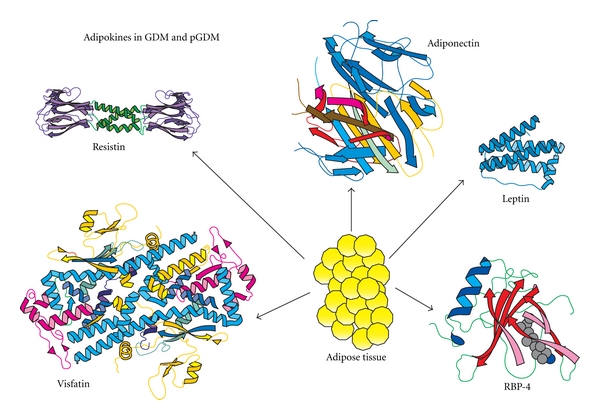
Molecular structure of the main adipokines produced by adipose tissue in GDM and pGDM. Adiponectin is a 244-amino-acid polypeptide that decreases as adipose tissue increases; it enhances insulin action and is decreased in pGDM and GDM. Leptin is a 167-amino-acid protein; it causes insulin action to increase and its levels show variability with respect to GDM and pGDM. Resistin (or adipose tissue-specific secretory factor) has 108 amino acids in the prepeptide form; its action on insulin has yet to be clarified. RBP-4 is a 183-amino-acid protein which reduces insulin action. Both resistin and RBP-4 levels appear increased, decreased, or unaltered in GDM but are uniformly increased in pGDM. The visfatin molecule consists of 491 amino acids; it facilitates insulin action and increases in GDM, while its actions in pGDM need further clarification.

Some studies have shown elevated levels in women with GDM [149–151] with a further increase of these levels detected in the presence of high maternal blood glucose levels. In contrast, a number of other studies found that visfatin levels are lower in GDM [146, 152–154].  Figure 1 displays aspects of the aforementioned adipokines.

## 3. Other Mediators of Inflammation in pGDM

### 3.1. TNF-*α* and IL-6

TNF-*α* and IL-6 are inflammatory mediators produced by monocytes and macrophages in the adipose tissue. These cytokines are increased in obesity and have multiple effects on insulin sensitivity in muscles, liver, or beta cells of the pancreas, ultimately leading to insulin resistance [32, 168]. In pregnancy, TNF-*α* and IL-6 production occurs in placenta [163, 164], while it is considered that a chronic inflammatory process in the adipose tissue may contribute to pregnancy-induced insulin resistance [164–167, 169]. Placental production of TNF has been shown to be maximized late in pregnancy [163] and to decline rapidly after pregnancy, this being in accordance with placental production of TNF-*α* [155]. In early pregnancy TNF-*α* mRNA is present mainly in syncytiotrophblast. Later in pregnancy and specifically near term, TNF-*α* mRNA also appears in villous stromal cells and TNF-*α* transcripts are present in maternal cells in the decidua adjacent to the extracellular membranes [164]. Most of the TNF-*α* produced by the placenta is delivered to maternal circulation and by comparison only a small amount to the fetal compartment [164].

Though in a GDM pregnancy IL-6 and TNF-*α* rarely increase [165–167], when this does occur, the increase is caused by the oxidative stress and the inflammation associated with the hyperglycemia [169]. Conversely, TNF-*α* possibly inhibits insulin secretion and insulin regulated glucose uptake in GDM [84, 104, 165, 166, 170]. An in vitro experiment showed that placentas from women with GDM released more TNF-*α* in response to a glucose stimulus than placentas from women with normal glucose tolerance [156].

IL-6 levels are significantly higher in women with pGDM at least 1 year postdelivery (mean 4 years postpartum) compared with normal controls [98], this difference becoming nonsignificant when it was adjusted for BMI. It is interesting to note that when nonobese women with pGDM were examined, the difference remained significant for IL-6 (BMI played no role) and that IL-6 was positively related to CRP, this persisting even after adjustment for BMI.

TNF-*α* was not significantly different in pGDM 3 months after delivery compared to women with normal glucose tolerance during pregnancy [43]. By contrast, IL-6 was significantly higher before adjusting for BFM but after adjustment for BFM adiponectin did not correlate to IL-6. Hauguel-de Mouzon et al. reported in a recent study that TNF-*α* rose significantly 6 weeks postpartum both in women with normal pregnancies and in GDM pregnancies when compared to antepartum values [100]. The increase for TNF-*α* was not significant for both groups 6 months postpartum. 

In another study, 18 women with pGDM at least 12 months after delivery were compared with normal controls and women with polycystic ovary syndrome (PCOS). They found no significant difference in TNF-*α* between the pGDM cases and controls, although the difference between PCOS and controls was significant [157]. 

### 3.2. C-Reactive Protein

CRP, an inflammatory agent common in numerous pathologic conditions, has been associated with metabolic abnormal states such as insulin resistance, hyperglycemia, and T2DM [31, 34–36], while it also appears to be associated with central obesity [44, 46].

In the first trimester of pregnancy the levels of CRP are increased and have been related to higher risk for GDM development [156, 157], this association also being valid with measurement of CRP later in the course of pregnancy [160, 170]. Furthermore, CRP is increased in maternal obesity, insulin resistance, and maternal dysglycemia [158, 161, 162]. The pathophysiologic role of inflammatory proteins and adiponectin seem to be the gradual impairment of beta cell function and increasing insulin resistance, which results in ineffective plasma glucose regulation and subsequent dysglycemia in the months and years that follow pregnancy.

As concerns pGDM, there is some evidence that women with a history of prior GDM have postpartum increased CRP that manifests chronic subclinical inflammation [40–46]. Increased CRP levels in women with pGDM, which have also been related to metabolic syndrome [45], have been reported in several studies [43–46].

The postpartum period which is complicated by gestational diabetes is a period of chronic subclinical inflammation. Some investigators have shown significantly increased levels of CRP 3 months after delivery in women with pGDM compared with controls [43]. Other authors also found a negative correlation between adiponectin levels and CRP, but this correlation became nonsignificant after adjustment for BFM, the latter being explained by the finding that CRP is also related to central obesity [44, 46]. The NHANES III study showed that adjustment of CRP values for waist circumference attenuated differences in women with pGDM and normal women [171].

Another study [172] recruiting 46 women with pGDM 3 years postpartum reported that women with pGDM had significantly elevated high-sensitivity CRP (hs-CRP) compared with controls.

Heitritter et al. [90] studied 25 women with pGDM and found that in a mean period of 4 years postpartum they had significantly higher CRP levels compared to controls. The difference remained significant after adjustment for BMI. CRP was negatively related to adiponectin and positively related to IL-6 and these associations remained unchanged after adjustment for BMI.

In another study, 18 women with pGDM at least 12 months after index delivery were found to have no significant difference in hs-CRP compared with normal controls [157].

On the other hand, other authors [46] studied 70 women with pGDM 6 years after their pregnancy and found significantly higher CRP levels in women with pGDM in the presence of abdominal obesity; they also found abnormal glucose tolerance compared with the women without a history of GDM. This was further confirmed in another study where significantly elevated CRP levels and fibrinogen were detected in 26 women with pGDM as compared with controls [44].

### 3.3. Plasminogen Activator Inhibitor 1

Plasminogen Activator Inhibitor 1 (PAI-1) is a protein that in humans is encoded by the SERPINE1 gene and is mainly produced by the endothelial cells, though it is also secreted by other tissue types, such as adipose tissue. Its main function is to inhibit tissue plasminogen activator (tPA) and urokinase (uPA), the activators of plasminogen, and hence fibrinolysis. PAI-1 is increased in various disease states, such as obesity, MS, insulin resistance, and T2DM [31, 34–36].

PAI-1 is increased in women with pGDM compared with normal women 3 months after delivery [43]. In this study PAI-1 remained higher after adjustment for BFM, while the authors also found a negative correlation between adiponectin levels and PAI-1.

Another study [172] reported that women with pGDM had significantly elevated PAI-1 compared with controls 3 years after delivery.

Other authors studying 74 women with pGDM 3 months after delivery found them to exhibit increased PAI-1 levels when they had impaired insulin sensitivity postpartum, while tPA was also observed to be elevated [42]. In another study, 25 women with pGDM demonstrated significantly higher PAI-1 levels compared to controls in a mean period of 4 years postpartum [98], although the difference lost significance after adjustment for BMI. Meanwhile, adiponectin levels correlated to PAI-1 levels before and after adjustment for BMI.

## 4. Conclusions

Women with pGDM are characterized by chronic subclinical inflammation which is associated with insulin resistance and abnormality in glucose metabolism. Approximately 30% of these women have metabolic syndrome and many of them will develop T2DM within 5 years of diagnosis [26, 31, 173]. The conversion rates from GDM to T2DM range from 2.6% to 70% over a period of 6 weeks to 28 years postpartum [174]. The problem of gestational diabetes is common and its incidence is exhibiting an increasing prevalence. Early recognition and management of women predisposed to develop T2DM later in their lives is thus crucial in the development of primary health care strategies, modification of lifestyle, and dietary habits significantly enabling prevention or delay of appearance of glucose intolerance states in predisposed women.

## Figures and Tables

**Table 1:** Adipokines and other inflammatory mediators in normal pregnancy, obesity/DM, GDM, and pGDM.

	Pregnancy	Obesity/DM	GDM	pGDM
Adiponectin [43, 50–92]	Decreased Unaltered	Decreased	Decreased	Decreased
Leptin [43, 91–105]	Increased	Increased	Increased Unaltered Decreased	Increased Unaltered
Resistin [68, 70, 73, 76, 106–121]	Increased	Increased	Increased Unaltered Decreased	Increased
RBP-4 [106, 122–138]	Increased Decreased	Increased Decreased	Increased Decreased Unaltered	Increased
Visfatin [139–154]	Increased	Increased	Increased	?
CRP [31, 34–36, 40–46, 90, 155–161]	Increased	Increased	Increased	Increased
PAI-1 [31, 34–36, 42, 43, 98, 162]	Increased	Increased	Increased	Increased
IL-6 [98, 163–167]	Increased	Increased	Increased	Increased
TNF-*α* [43, 84, 100, 103, 104, 155, 163–167]	Increased	Increased	Increased	Increased
